# An Updated Overview of Cyclodextrin-Based Drug Delivery Systems for Cancer Therapy

**DOI:** 10.3390/pharmaceutics14081748

**Published:** 2022-08-22

**Authors:** Dan Nicolae Păduraru, Adelina-Gabriela Niculescu, Alexandra Bolocan, Octavian Andronic, Alexandru Mihai Grumezescu, Rodica Bîrlă

**Affiliations:** 1 “Carol Davila” University of Medicine and Pharmacy, 050474 Bucharest, Romania; 2 University Emergency Hospital of Bucharest, 050098 Bucharest, Romania; 3 Department of Science and Engineering of Oxide Materials and Nanomaterials, Faculty of Applied Chemistry and Materials Science, Politehnica University of Bucharest, 011061 Bucharest, Romania; 4 Research Institute of the University of Bucharest—ICUB, University of Bucharest, 050657 Bucharest, Romania; 5 Academy of Romanian Scientists, Ilfov No. 3, 050044 Bucharest, Romania; 6 Saint Mary Clinical Hospital, 011172 Bucharest, Romania

**Keywords:** cyclodextrins, delivery systems, cancer treatment, anti-cancer drug formulations

## Abstract

Encompassing a group of complex and heterogeneous diseases, cancer continues to be a challenge for patients and healthcare systems worldwide. Thus, it is of vital importance to develop advanced treatment strategies that could reduce the trends of cancer-associated morbidity and mortality rates. Scientists have focused on creating performant delivery vehicles for anti-cancer agents. Among the possible materials, cyclodextrins (CDs) attracted increasing interest over the past few years, leading to the emergence of promising anti-tumor nanomedicines. Tackling their advantageous chemical structure, ease of modification, natural origin, biocompatibility, low immunogenicity, and commercial availability, researchers investigated CD-based therapeutical formulations against many types of cancer. In this respect, in this paper, we briefly present the properties of interest of CDs for designing performant nanocarriers, further reviewing some of the most recent potential applications of CD-based delivery systems in cancer management.

## 1. Introduction

Cancer is one of the top 10 leading causes of death worldwide, posing a serious threat to human health and impeding the increase in life expectancy in every country of the world [1,2,3,4,5,6,7,8]. Particularly, diagnosing cancers in terminal phases leaves patients with very few and rather inefficient treatment options [4,9,10]. Even though the diversity of cancers gives clues on its underlying causes, it also highlights the need for intensifying research efforts toward finding better ways to control the disease [1].

Various drugs have been involved in the treatment of cancer, with different degrees of efficacy. Despite their promising anti-cancer activity, traditional drugs often lack the required physicochemical properties for being performant medicines against the disease. Specifically, they exhibit poor water solubility, instability, short drug cycle time, poor permeability, and low biocompatibility, making it hard for the substances to reach the desired site at an optimum concentration in a safe manner for the healthy tissues [3,4,11,12,13,14]. Thus, it became imperative to create efficient drug delivery vehicles that would carry anti-cancer agents to tumor cells with improved therapeutic efficacy and minimal toxicity to normal cells. In this respect, a wide range of materials aroused recent scientific interest in creating targeted biocompatible nanocarriers for anti-cancer drugs [4,13,15,16,17,18,19,20,21].

One particularly appealing option for encapsulating anti-cancer agents is represented by cyclodextrins (CDs). These compounds are natural cyclic oligomers with well-proved utility in drug delivery. CDs can be obtained from the enzymatic hydrolysis of starch and admit facile chemical modification. Moreover, their commercial availability, low immunogenicity, biocompatibility, and safety are important advantages for incorporating CDs in pharmaceutical formulations of need in cancer therapy. A peculiar beneficial characteristic of CDs for drug encapsulation resides in their structure, as CDs have a hydrophilic outer surface and a lipophilic central cavity that allows the formation of host–guest complexes with fitted molecules [2,3,4,22,23,24,25].

With these advantages in mind, researchers employed CDs in developing numerous innovative drug delivery vehicles for anti-cancer drugs. In this context, we briefly elaborate on the properties of CDs for creating performant nanocarriers, further reviewing the newly envisioned CD-based anti-cancer formulations according to the type of cancer they aim to treat.

## 2. Cyclodextrins—Properties of Interest for Creating Performant Nanocarriers

**Figure 1:**
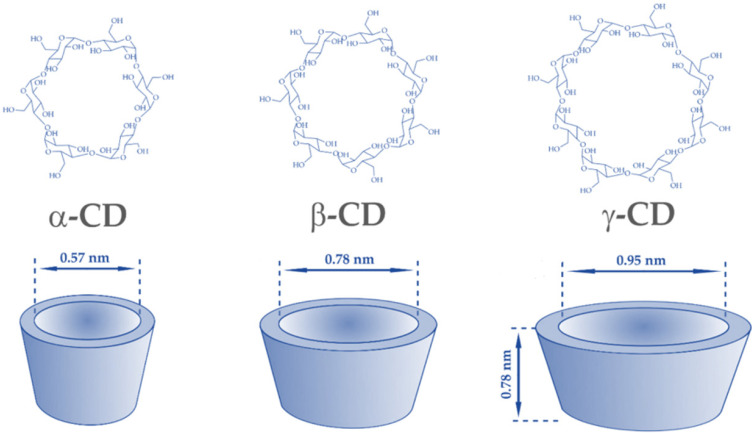
α-, β-, and γ-CD (cyclodextrin) molecules. Adapted from an open-access source [27].

CDs are a class of cyclic oligosaccharides presenting six, seven, or eight glucose units linked by α-1,4 glycosidic bonds, which are called α, β, and γ-CD, respectively [26,27] (Figure 1). Given the chair structure of the glucopyranose groups, CDs’ shape is that of a hollow truncated cone, with a hydrophobic cavity and a hydrophilic external surface. This structural arrangement allows for the encapsulation of hydrophobic drugs into CD’s cavity, creating host–guest complexes without implying sophisticated chemical reactions [4,27,28,29]. Moreover, it is possible to form reversible inclusion complexes between CDs and various guest molecules, thus making it convenient not only for drug loading but also for drug release at the desired site [23,30,31].

Complex formation occurs when a dimensional fit between the host cavity and guest molecule is met. Regarding the structure of the guest molecules, they can include both hydrophilic and hydrophobic linear or cross-linked functional groups in their structure. However, only the hydrophobic parts with smaller characteristic dimensions than the inner diameter of the inner cavity can be absorbed inside [32,33,34].

**Figure 2:**
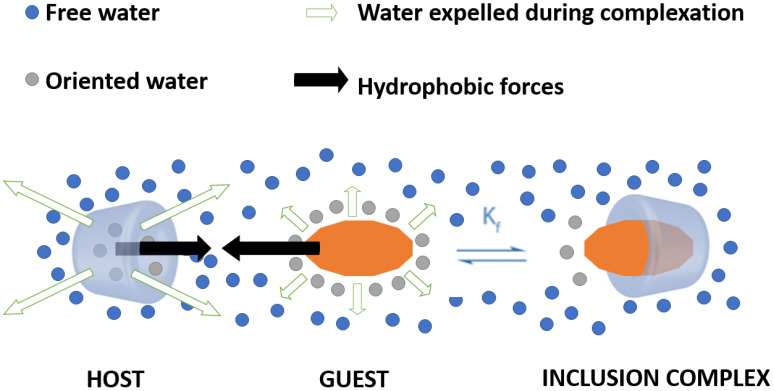
Schematic representation of the formation of an inclusion complex between a CD (host) and a guest.

The formation of inclusion complexes is achieved under a dynamic association/dissociation equilibrium between free guest molecules, un-complexed CDs, and the complex [35,36], the direction of this reversible process being decided by the Kf constant (formation/stability constant) (Figure 2). Specifically, the higher the Kf value, the more stable is the complex, and hence less prone to dissociation [37].

Complexation is considered an entropy-driven process as its main driving force is the hydrophobic effect (i.e., the release of enthalpy-rich water molecules from CDs cavity). Moreover, complex formation is also influenced by other forces, including complementary interactions (e.g., van der Waals forces, hydrogen bonds, electrostatic interactions, hydrophobic interactions) and the release of conformational strain [37,38,39].

In more detail, water molecules are displaced by the more lipophilic chemical entities present in the solution to attain apolar–apolar association. The effect is the decrease in CD cycle strain, hence a more stable lower energy state of the complex. The cause behind this process is that pure water molecules adopt an entropy-maximizing structure, owing to the formation of a dynamic 3D hydrogen bonding network, which is partially disrupted around the nonpolar solute. Therefore, the hydrophobic molecule is caged in a clathrate-like basket shape, where the H-bonds are reoriented tangentially to such a surface to minimize the number of disrupted physical bonds. To maximize the entropy of the system, water molecules are expelled from the CD cavity, whereas the hydrophobic molecule shifts inside the CD cavity to form the inclusion complex [34,40].

Thus, when designing CD-based delivery systems, it is essential to consider their physicochemical properties to ensure a proper fit between the host and guest molecules. In this respect, some of the most important properties of native CDs have been gathered in Table 1.

Another important property of CDs is their lack of toxicity to humans, which allowed for the approval of the Food and Drug Administration (FDA) and the European Medicines Agency (EMA) for use in pharmaceutical formulations. Therefore, they are safe to use as excipients for increasing the stability and bioavailability of poorly hydrophilic compounds [28,44]. Other beneficial effects on guest drugs include prolongation of the shelf life of drugs, reduction (or elimination) of unpleasant taste and odor, and prevention of drug–drug or drug–excipient interaction [33,45,46]. Delivering drugs within the cavity of CDs can also alleviate their adverse side effects to some extent [4], especially due to their ability of controlled release.

In general, the mechanism of controlled degradation of inclusion complexes is based on a change in pH, which leads to the loss of hydrogen between the host and the guest compounds. An alternative disassembly method involves heating or enzymatic cleavage of α-1,4 links between glucose units [40]. For inclusion complexes containing multiple guest components or different cyclodextrin types, the guests are not necessarily released in the same ratio as in the original (pre-formation) guest mixture. Each complex has a different solubility and a different rate of release. Therefore, it is also possible to attain an intended release pattern by alteration of the guest formulation [33].

In addition, CDs can be easily functionalized, an important property for designing targeted delivery systems. In more detail, targeting ligands can be attached to CDs, realizing vehicles that can benefit from the strong binding ability to target ligands and receptors to reach the surface of tumor cells, where some receptors (e.g., folate receptor, biotin receptor, glucose receptor) are overexpressed [4,47,48].

## 3. Cyclodextrins as Carriers of Anti-Cancer Drugs

**Figure 3:**
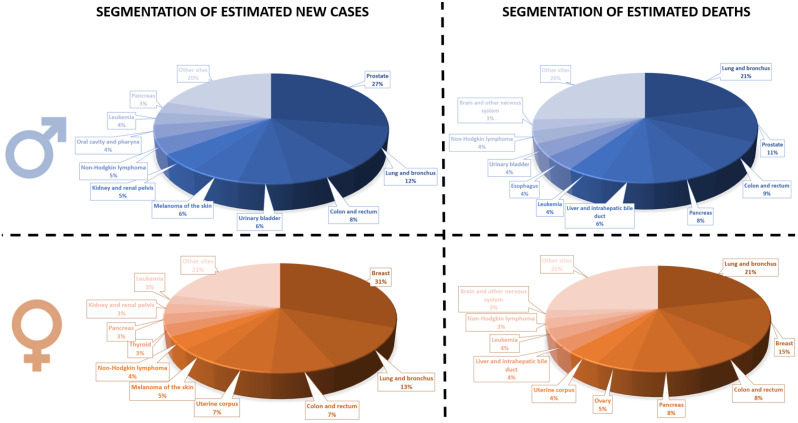
Ten leading cancer types for the estimated new cancer cases and deaths by sex, United States, 2022. Created based on information from [51].

Cancer denotes a group of more than a hundred types of diseases characterized by the malignant form of abnormal tissue growth in different body parts (Figure 3). As one of the leading causes of morbidity and mortality worldwide, cancer remains an ongoing challenge for the healthcare system, requiring prompt and adequate treatment to prevent severe health problems, prolong patients’ survival rates, and improve their life quality [21,49,50].

The most common strategy in fighting cancer is chemotherapy. Depending on the cancer stage, it can be used alone or in combinatorial approaches with radiotherapy, surgery, or adjuvant therapies (e.g., immunotherapy, hormone therapy, photothermal therapy, photodynamic therapy, ablative techniques) to produce effective anti-tumor responses [18,52,53,54,55]. Nonetheless, despite the existence and clinical use of several chemotherapeutic drugs, their application is restricted due to unfavorable physicochemical properties (e.g., poor aqueous solubility, photolability, unpleasant smell) and hard-to-maintain balance between efficacy and systemic adverse side effects [3,56,57,58]. A growing number of chemotherapeutic agents have been developed in the past few decades. However, they did not conquer the market and clinical settings because of their severe toxicity, high production cost, and low patient compliance [18,57].

Therefore, recent research efforts have focused on developing drug delivery systems able to circumvent these limitations and improve the drug’s therapeutic potential [21,58,59]. In this regard, the use of nanotechnological approaches has appeared as a viable solution for improving the solubility and bioavailability of anti-cancer compounds of both natural and synthetic origin and for delivering them across biological barriers (e.g., tumor microenvironment and vasculature, reticuloendothelial system, blood–brain barrier) [18,19,20,60].

Thus, having the necessary characteristics to overcome the drawbacks of traditional chemotherapy drugs, CDs have been investigated as carriers for the delivery of various cargos to different tumor types [3,13,61,62,63]. In this respect, the following subsections describe some of the newest advancements in the CD-based drug delivery systems that hold promise in treating the most common or deadly types of cancer.

### 3.1. Lung Cancer

Lung cancer represents one of the most commonly diagnosed types of cancer worldwide. Despite medical progress in recent years that led to a decrease in lung cancer incidence, it remains the deadliest type of cancer, being associated with very low survival rates at mostly all stages. Thus, research efforts continue to be directed toward finding better treatment alternatives for lung cancer [22,51,64,65,66]. In this respect, a series of studies in the field focused on improving chemotherapeutic and photodynamic approaches by incorporating CDs in the therapeutic formulation.

Dai et al. [67] have developed CD prodrug supramolecular nanoparticles containing a reduction-sensitive disulfide bond-linked permethyl-β-CD-Camptothecin prodrug, water-soluble adamantane–porphyrin photosensitizer, hyaluronic acid grafted by triphenylphosphine, and β-CD through an orthogonal host–guest recognition strategy. The as-designed system could be easily taken up by mitochondria of A549 cancer cells, release the active anti-cancer agent in situ, and produce reactive oxygen species (ROS) under light irradiation through its porphyrin component. Therefore, these nanoassemblies represent an efficient approach against lung cancer through their two-step synergistic chemo-photodynamic effects.

Alternatively, Guimares et al. [68] have complexed with CDs a novel orally bioavailable cancer therapeutics under clinical testing, namely, the porcupine inhibitor LGK974. These complexes enabled safer and repeated oral or parenteral administration of LGK974, increasing its solubility and bioavailability while decreasing its toxicity in tissues that rely on Wnt signaling for normal homeostasis.

A different drug delivery option is offered by Vaidya et al. [69], who loaded erlotinib into β-CD and coated the resulting structures with poly(lactic-co-glycolic acid). These nanoparticles significantly improved therapeutic efficacy against non-small cell lung cancer (NSCLC) cells, lowering IC_50_ values, suppressing colony forming ability of tumor cells, enhancing apoptosis, and inhibiting autophagy.

With a similar purpose in mind, i.e., to find better treatment strategies for NSCLC, Wang and colleagues [70] complexed resveratrol with sulfobutylether-β-CD, and loaded the constructs onto polymeric nanoparticles. Compared to free anti-cancer agents, the nanoplatforms exhibited better therapeutic efficacy. Specifically, the designed drug delivery systems increased cellular uptake, cytotoxicity, and apoptosis, also retaining resveratrol’s antioxidant properties.

The same carrier was chosen by Shukla et al. [71] for the delivery of Celastrol. The complexation with the CD derivative improved the drug’s permeability across the intestinal tissue, increased its stability at physiological pH, and enhanced the cytotoxic efficacy in human lung cells.

A recent study conducted by Lin et al. [72] investigated the administration of metformin in patients with lung cancer and type 2 diabetes. The researchers used a carrier system consisting of β-CD polycaprolactone block copolymers conjugated with folic acid. The delivery platform endowed the drug with excellent stability, controlled release, and targeting ability. In more detail, metformin release was significantly faster at a pH of 6.4 than at physiological pH, while the match between folic acid ligands and folate receptors ensured cellular internalization via endocytosis in A549 lung tumor cells. Thus, due to controllable release and active targeting, the nanosystem provides promising anti-tumor efficacy with minimum toxicity toward normal cells.

### 3.2. Gastrointestinal Cancers

Taken together, gastrointestinal tract cancers account for more than a quarter of the total cancer-associated deaths worldwide [51,73], imposing an urgent need for developing more performant treatments. Thus, the use of CDs was also exploited for developing novel drug formulations for cancers of the digestive system [74].

For instance, Catchpole et al. [75] have created complexes between New Zealand propolis and γCD, αCD, and βCD that inhibited the proliferation of four human gastrointestinal cancer cell lines (i.e., DLD-1, HCT-116, NCI-N87, KYSE-30). The developed complexes also exhibited strong anti-inflammatory and lipid antioxidant activities. The effects of these complexes are attributed not only to the activity of encapsulated propolis components (i.e., flavonoids, phenolic acids, caffeate-type esters), but also to the synergism between CDs and these bioactive compounds.

A recent study conducted by Altoom et al. [76] focused on encapsulating oxaliplatin into a β-CD/phillipsite (β-CD/Ph) composite. The system represents an advanced, low-cost, effective option for delivering the drug to cancer cells. The nanocarrier presented significant activity for the composite itself while also enhancing the effect of the loaded drug on colorectal cancer cells (HCT-116). Furthermore, the composite was reported to be safe and biocompatible when tested on normal colorectal cells (CCD-18Co).

Al-Abboodi et al. [77] used a CD derivative (i.e., hydroxypropyl-β-CD) as a host for guest anti-cancer drug Clausenidin. The inclusion complex allowed for constant drug release with time, increased drug solubility, and displayed a greater cytotoxic effect on colon cancer HT29 cells than the drug alone. The action mechanism of the complexes resides in triggering ROS-mediated cytotoxicity in cancer cells, inducing cell cycle arrest and death by apoptosis associated with caspases activation. Moreover, a reduction in the side effects was noticed by comparing cell viability between normal and cancer cells.

Elamin et al. [78] have also employed a CD derivative-based nanomedicine as an anti-cancer strategy. The authors created a folate-appended methyl-β-CD (FA-M-β-CyD) complexed with adamantane-grafted hyaluronic acid. The supramolecular complex exhibited higher cytotoxic activity in HCT116 cells than FA-M-β-CyD alone, as this cancer cell line expresses both folate receptor-α and CD44. Thus, this dual-targeting system holds promise for creating tumor-selective agents for treating colorectal cancer.

Alternatively, Akkın et al. [79] prepared a CD nanoplex based on charge interaction to deliver the anti-cancer drug 5-fluorouracil and Interleukin-2 as a chemo-immunotherapy strategy against colorectal cancers. Drug-loaded nanoplexes displayed higher cytotoxicity than free drug solution against CT26 mouse colon carcinoma cells. The delivery system was also considered promising due to the absorptive/cellular uptake effect of CDs, the effective co-transport of chemotherapeutic drugs and immunotherapeutic molecules, and the possibility of reducing or avoiding the toxicity of carried agents.

Sun et al. [80] recently created a folate-targeted polyethylene glycol-modified amphiphilic CD nanoparticle for co-encapsulation of ginsenoside Rg3 and quercetin. The nanocarrier significantly prolonged blood circulation and improved tumor targeting in an orthotopic colorectal cancer mouse model. Moreover, the system could considerably improve the survival time of animals when combined with Anti-PD-L1.

A similar functionalization approach was employed by Zou and colleagues [81]. The researchers developed an amphiphilic cationic CD nanoparticle modified with PEGylated folate for the co-delivery of docetaxel and siRNA. The nanoformulation achieved colorectal cancer cell-specific uptake due to folate targeting ligand incorporation. Furthermore, the nanosystem augmented the apoptotic effect of the drug with the downregulation of RelA expression, retarding tumor growth without causing significant toxicity.

Other CD-based nanosystems were proposed by Ünal et al. [82], who designed two different amphiphilic CDs coated with polyethyleneimine or chitosan for the delivery of camptothecin. The nanosystems proved highly cytotoxic on HT29 cells in comparison to equivalent camptothecin in solution. Moreover, studies on Caco-2 cells showed that the cationic CD nanoparticles lead to a 276% increase in drug permeability and significantly higher mucosal penetration.

On a different note, Zhang et al. [83] fabricated a supramolecular system [Pt(IV)-SSNPs] based on poly(β-CD) for delivering adamantly functionalized platinum(IV) prodrug [Pt(IV)-ADA2] to CT26 cells. The prodrug can be converted to active cisplatin in the tumor environment, thus reducing systemic toxicity. The host–guest complexes were successfully uptaken by targeted cells, leading to cell cycle arrest in the G2/M and S phases and inducing apoptosis. Given the effective tumor accumulation, enhanced anti-tumor effect, and negligible cytotoxicity to major organs, the authors concluded that these nanoparticles represent promising candidates for efficient cisplatin delivery and cancer treatment.

As liver cancer represents the sixth most diagnosed cancer worldwide and one of the top three most common causes of cancer death [84], it is no surprise that several studies have also focused on designing CD-based nanomedicines for this type of cancer. Aiming to create a targeted delivery vehicle for doxorubicin, Fan and colleagues [85] have fabricated folic acid–polyethylene glycol–β-CD nanoparticles. The nanocarrier improved drug solubility and controlled its release without inducing blood hemolysis, thus being a potential platform for liver cancer therapy.

Differently, Yang et al. [86] synthesized a targeted prodrug based on a host–guest complex containing β-CD as the host molecule, benzimidazole for pH sensitivity, lactobionic acid coupled with polyethylene glycol as targeting moieties, and doxorubicin as the guest molecule. The pH-sensitive supramolecular assembly enabled the accelerated release of encapsulated drugs with the decrease in pH value. Moreover, when taken up by HepG2 cells, the complexes proved highly cytotoxic, inhibiting liver cell proliferation through apoptosis induction.

### 3.3. Breast Cancer

Breast cancer is the first type of cancer in terms of incidence among women worldwide and holds the second rank of associated mortality in the USA [51,87,88]. The first treatment option combines the use of chemotherapeutic agents, radiation therapy, and surgical intervention. However, anti-cancer drugs do not yet penetrate the tumor cells in adequate concentrations, leading to systemic side effects and reduced pharmacokinetics [89,90]. Therefore, scientists have put effort into designing nanomaterials that would facilitate breast cell targeting and drug internalization.

For instance, Hyun and colleagues [91] prepared a nanocarrier composed of β-CD, polyethylene glycol, and folic acid for the delivery of doxorubicin. When administered intravenously to test animals, the complexes decreased tumor volume without imparting systemic toxicity and cardiotoxicity. Thus, the designed system can maximize the efficacy of doxorubicin delivery in a safe, targeted manner.

Another delivery strategy for breast cancer was proposed by Farrokhi et al. [92]. The authors created a β-CD polymer nanocarrier to deliver RNA-cleaving DNAzyme targeting c-Myc gene in MCF-7 cell line. Test results showed that this delivery system had a synergistic effect in combination with doxorubicin, leading to stronger inhibitory activity against breast cancer cell proliferation.

Differently, Mihanfar et al. [93] included doxorubicin into a targeted delivery carrier of β-CD-functionalized dendrimeric graphene oxide magnetic nanoparticles. In this manner, drug efficacy was increased through more pronounced cellular proliferation inhibition and apoptosis induction. Moreover, the nanocarrier reduced off-target side effects of doxorubicin in vivo. The authors also considered that graphene oxide nanoparticles could sensitize breast cancer cells to the anti-cancer agents, yet further in vivo testing should be carried out to assess this hypothesis.

Zafar et al. [94] have also tackled the benefits of β-CDs, complexing them with genistein and D-α-Tocopherol polyethylene glycol 1000 succinate. Including the drug in these complexes resulted in its solubility enhancement that further led to significantly greater antioxidant and cytotoxic activities compared to pure genistein.

Alternatively, Lee et al. [95] combined β-CD, polyethylene glycol, and folic acid to create an advanced nanocarrier for adamantane and near-infrared fluorophore (NIRF) conjugate to the breast tumor target site. The delivery system proved to be highly efficient, showing excellent tumor targetability.

A recent study conducted by Panagiotakis and colleagues [96] described the incorporation of water-insoluble photosensitizers (i.e., meso-tetraphenylporphyrin and meso-tetra(m-hydroxyphenyl)porphyrin) into permethyl-β-CD. The complexes exhibited favorable properties when incubated with MCF-7 cells, such as photostability, intense intracellular fluorescence, high photokilling efficiency, and low dark toxicity.

Soleimani et al. [97] have developed a different nanocarrier. The authors prepared a magnetic drug delivery system based on β-CD and poly(2-ethyl-2-oxazoline) embedded with iron oxide nanoparticles and loaded with doxorubicin hydrochloride. The developed magnetic nanogel provided high drug loading, slow and stimuli-triggered drug release, and excellent cytocompatibility. Moreover, it allowed efficient dual cancer therapy, combining the benefits of chemotherapy and hyperthermia therapy.

On a different note, Ercan et al. [98] created blank 6OCaproβCD nanoparticles and administered these constructs to MCF-7 cells. Studies revealed increased levels of apoptosis-related proteins without affecting HDGF protein level, emphasizing the ability of designed nanoparticles to prevent cell proliferation. Thus, given their intrinsic apoptotic effect, these vehicles represent promising nanocarriers for chemotherapeutic drugs in treating breast cancer.

Another β-CD-based nanosystem is proposed by Kasinathan et al. [99]. The authors prepared a hybrid nanocomposite made of β-CD and molybdenum disulfide that showed remarkable antibacterial properties and potent cell inhibition activity against MCF-7 breast cancer cells. Thus, it can be considered a promising material for cancer therapy.

### 3.4. Reproductive Cancers

Cancers of the reproductive system also pose an important burden on both males and females worldwide, being among the top ten types of cancers in terms of incidence and mortality [51,100]. In this respect, CD-based formulations aim to offer better therapeutic alternatives, being researched for the treatment of cancers of the prostate, cervix, and ovaries.

An example of a CD-based delivery system for prostate cancer is offered by Baskar and Supria Sree [101]. The authors incorporated l-asparaginase into a β-CD–chitosan nanobiocomposite, obtaining good anti-cancer activity against prostate cancer cell lines with an IC_50_ value of 125 µg/mL, which is half the concentration needed for the free enzyme. Alternatively, Trindade et al. [102] formed inclusion complexes between β-CD and carvacrol. These nanoconstructs led to a dose-dependent inhibition of tumor cells in 2D and 3D cell culture systems, having potent anti-proliferative effects against PC3 cells in vitro.

Progress has also been noted in developing therapeutic formulations for cervical cancer. Kost et al. [103] proposed an example of a CD-based delivery system. The authors created a β-CD core complexed with doxorubicin and loaded it into stereocomplexed micelles (SCMs) made of polylactides. The system offered a controlled drug release and a more efficient tumor cell suppression compared to free drugs. A different nanoconstruct was designed by Reis and colleagues [104], who prepared gold-core silica shell (AuMSS) nanoparticles coated with poly-2-ethyl-2-oxazoline (PEOZ) and β-CD in different ratios. The coatings endowed the nanomaterials with better biological performance, enhanced cytocompatibility, and increased internalization rate by the HeLa cancer cells.

A promising solution has also been proposed by Russo Spena et al. [105] for ovarian cancer therapy. The researchers encapsulated a Pin1 inhibitor in modified CDs and remotely loaded it into pegylated liposomes. The as-designed nanoconstructs preferentially accumulated at the tumor site and showed a desirable pharmacokinetic profile. The carried drug managed to alter Pin1 cancer-driving pathways by inducing proteasome-dependent degradation of Pin1, the delivery system as a whole proving to be an effective alternative for curbing ovarian tumor growth in vivo.

### 3.5. Kidney Cancer

Kidney cancer represents another malignant disease with high morbidity and mortality rates worldwide [51,106,107], and has attracted scientific interest towards creating advanced drug delivery systems for achieving better therapeutic outcomes.

Bomzan et al. [108] recently developed a complex of ticlopidine hydrochloride (TCP) with β-CD that helped improve the thermal stability of the drug. Moreover, the system demonstrated significant in vitro cytotoxicity compared to pure TCP towards human kidney cancer cell line (ACHN) due to a more pronounced ROS generation and increased apoptotic activity.

A different inclusion complex is proposed by Han and colleagues [109], who prepared a taccalonolide AJ-hydroxypropyl-β-CD formulation. The designed drug delivery system possessed superior solubility, stability, selectivity on the clear-cell renal cell carcinoma cell line 786-O vs. normal kidney, and anti-tumor activity than the free steroid. The underlying mechanisms for the anti-cancer activity consisted of arresting the target cells in the G2/M phase and inducing cell apoptosis by disrupting microtubule dynamics. Furthermore, the inclusion complex enhanced the therapeutic window and reduced toxicity by changing the injection time.

### 3.6. Brain Cancer

For brain cancers, drug delivery faces additional difficulties attributed to the complexity of the brain and the presence of the blood–brain barrier (BBB). As the BBB impedes therapeutics to enter the brain, only a small portion of the administered medicine can reach the target site, thus requiring higher doses that bring subsequential unwanted side effects. To overcome these challenges, nanomaterials may be used to mask the BBB-limiting properties of the drugs, allowing for safer drug dosing while achieving the desired anti-tumor efficacy [110,111,112,113,114].

In this context, Chen et al. [115] created a nanoparticle, called IT-101, formed by self-assembly of Camptothecin-conjugated CD-based polymers. A boost injection of this formulation could regulate NK cell activation and T cell proliferation, while the presence of CD-based polymer could facilitate the drug to inhibit tumor growth at an early stage. IT-101 delivered the drug close to the brain, reducing the potential adverse effects on peripheral tissues and increasing Camptothecin bioavailability in the brain.

A different brain delivery strategy was approached by Lin and colleagues [116]. The authors used CD-encapsulated butylidenephthalide in liposomal formulations. The system improved the bioavailability and stability of the drug, offering successful delivery to the brain and sustained release.

Alternatively, Qu et al. [117] created an inclusion complex between hydroxypropyl-β-CD and disulfiram to enhance the solubility, anti-glioblastoma activity, and safety of this anti-tumor drug. Administered by the intranasal route in combination with copper, this complex led to the inhibition of tumor growth and migration, stimulated apoptosis, and prolonged the survival time of tested animals.

### 3.7. Bone Cancer

Bone cancers account for less than 1% of all diagnosed cancers each year, yet they are associated with significant morbidity and mortality [118]. A common challenge in treating bone tumors is acquiring resistance to anti-cancer drugs. Hence, considerable attention from scientists revolves around developing delivery systems for improving therapeutic outcomes. In particular, the employment of bone-targeted nanocarrier systems has the potential to avoid using large doses of plain chemotherapeutics, concentrate drugs at the tumor site, protect therapeutic agents from rapid clearance, and reduce systemic adverse effects [119].

In this respect, Ahmadi et al. [120] have developed a smart nanosystem for targeted delivery of methotrexate for Saos-2 cell line. The nanocarrier consisted of a synthetic polymer with cationic moieties coating magnetic nanoparticles with CD anchoring sections. The as-designed delivery system allowed for pH-responsive drug release, with no substantial cytotoxicity on healthy cells. Moreover, the nanosystem exhibited successful tumor cell internalization, leading to a stronger anti-cancer activity on Saos-2 cells than free methotrexate.

Alternatively, Khelghati et al. [121] used a pH-sensitive magnetic hyperbranched β-CD nanocarrier for the delivery of doxorubicin to bone cancer cells. The nanoplatform proved completely biocompatible and displayed a better drug release in acidic pH values. The researchers concluded that this formulation has more cytotoxicity than the free anti-cancer drug after incubation time, showing high potential for targeted delivery to Saos-2 cell line.

Another example of a CD-based targeted delivery system is offered by Plesselova et al. [122]. The authors used polyethyleneimine as a polymeric scaffold for conjugating bisphosphonates as targeting ligands and CDs as supramolecular drug carriers. This delivery approach showed promising efficiency in delivering doxorubicin to bone cancer cells and metastases. In addition, this multicomponent nanosystem could be tailored ad hoc to various therapeutic strategies due to the adaptability of the ligand module and the CD drug tandem.

### 3.8. Other Anti-Cancer Applications of Cyclodextrin-Based Delivery Systems

In addition to the above-described CD-based delivery systems, several other studies have investigated the use of these versatile oligosaccharides for less explored types of cancers or for less specific/multiple applications.

For example, Bognanni et al. [16] developed cross-linked β-CD, γ-CD, and γ-CD functionalized with arginine–glycine–aspartic or arginine moieties nanocarriers for doxorubicin and oxaliplatin, testing their anti-proliferative activity against A549 and HepG2 cell lines. The CD polymers significantly increased the anti-tumor activity of carried drugs, holding promise for improving chemotherapeutic approaches. Specifically, a considerable improvement in the anti-tumor activity was noticed for nanosystems carrying doxorubicin in HepG2 cell lines, while delivery vehicles loaded with oxaliplatin enhanced anti-proliferative effects in both tested cell lines.

Alternatively, Wu and colleagues [123] created light/redox dual-stimuli responsive β-CD-gated mesoporous silica nanoparticles functionalized with an azobenzene/galactose-grafted polymer for the delivery of doxorubicin. The as-designed system showed an efficient delivery to HepG2 cells and displayed enhanced cytotoxicity in this cell line compared to HeLa and COS7 cells.

Another redox sensitive system was recently elaborated by Mousazadeh et al. [124], who used folate-appended polyethylenimine-β-CD host–guest supramolecular nanoparticles for the co-delivery of adamantane-conjugated doxorubicin and human telomerase reverse transcriptase–small interfering RNA (hTERT-siRNA). These inclusion complexes allowed a pH-dependent intracellular drug release in a sustained manner and effective gene transfection. Moreover, the system possessed excellent biocompatibility and hemocompatibility, while providing effective cell apoptosis of targeted folate receptor-positive cells.

A tumor microenvironment triggered nanoplatform was also developed by Jia et al. [125]. Specifically, the authors fabricated a highly sensitive ROS-responsive polymer (i.e., MPEG-CD-PHB) delivery vehicle for doxorubicin and purpurin 18. The nanocarrier allowed for controlled drug delivery, enabling enhanced anti-cancer activity through the collaborative effects of chemotherapy and photodynamic therapy.

On a different note, Pooresmaeil and Namazi [126] fabricated a β-CD-grafted magnetic graphene oxide-based delivery system with tumor cell-targeting ability. The nanocarrier was further evaluated for the delivery of doxorubicin and methotrexate, the results indicating a better drug release behavior in cancer cells than in healthy cells. Thus, the targeted system is both a safe and effective alternative to free drug administration.

Vukic et al. [127] prepared an acetylshikonin/β-CD inclusion complex and tested it against HCT-116 and MDA-MB-231 cell lines. Compared to the free cytotoxic agent, the complex exhibited a stronger short-term effect on HCT-116 cell, superior long-term effect on both cell lines, more pronounced cell cycle arrest and autophagy inhibition, and higher accumulation of intracellular ROS.

Hu and colleagues [128] created inclusion complexes between Saikosaponin-d (SSD) with hydroxypropyl-β-CD with application in skin cancer. The nanostructures presented enhanced aqueous solubility attributed to the transformation of crystalline structure to amorphous form and formation of hydrogen bonds between SSD and host CD. In addition, the delivery system induced apoptosis in HSC-1 cells through the activation of MAPK and suppression of Akt-mTOR signaling pathways.

A β-CD derivative was also employed by Parvathaneni and colleagues [129]. The authors have developed an inclusion complex between sulfobutyl ether β-CD and afatinib, to enhance its bioactivity and reduce p-glycoprotein efflux towards improving its transport across the intestinal membrane. The loading’s cytotoxicity increased after complexation across multiple cancer cell lines, the nanosystem being able to enhance drug’s tumor volume retraction capacity.

Differently, Xu et al. [130] compared a series of CDs for the encapsulation of NLG919. Out of the tested CDs, hyperbranched-β-CD was reported to be the most conducive for NLG919 solubilization and intravenous dosing. Furthermore, this complex could significantly enhance cytotoxic activity of paclitaxel consistent with suppression of IDO-1 mediated chemotherapy activation of tumor-targeted immune responses.

Rodell et al. [131] alternatively used β-CD nanoparticles as delivery vehicles for R848, an agonist of the toll-like receptors TLR7 and TLR8. The designed system was administered in multiple tumor models in mice and was observed to alter the functional orientation of the tumor immune microenvironment towards an M1 phenotype, resulting in controlled tumor growth and avoidance of tumor rechallenge. Moreover, in combination with the immune checkpoint inhibitor anti-PD-1, this novel treatment approach improved immunotherapy response rates.

A different strategy was employed by Zhang et al. [132]. The researchers designed an amphiphilic and cationic single-isomer CD for the delivery of chlorin e6. The nanosystem was noted to improve photodynamic therapy efficacy, exhibiting favorable stability, preferential cellular uptake, enhanced ROS generation, improved cell viability, and increased phototoxicity.

## 4. Discussion

**Figure 4:**
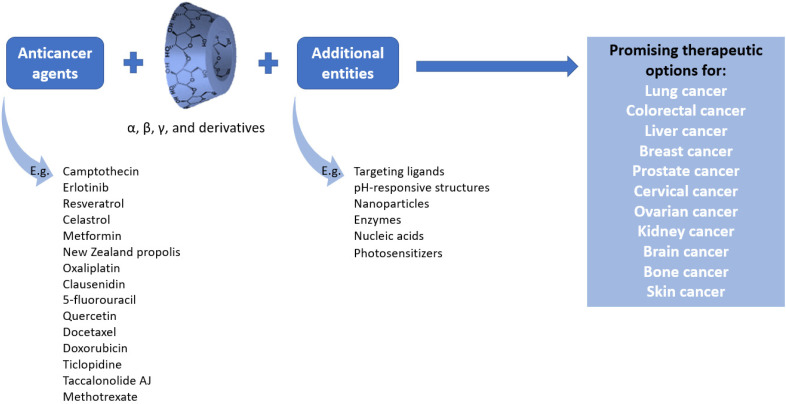
Overview of the main discussed possibilities of using CDs for the delivery of anti-cancer agents.

The advantageous properties of CDs for creating inclusion complexes with drugs have rendered them attractive for finding better anti-cancer chemotherapeutic strategies (Figure 4). Therefore, numerous studies have investigated the incorporation of conventional drugs or natural bioactive molecules into CD cavities and studied their effects against various cancer cell lines in vitro or as in vivo treatment approaches in animal models.

Native CDs (i.e., α-CD, β-CD, and γ-CD), their various derivatives, and different combinations with polymers, metal and metal oxide nanoparticles, liposomes, and micelles have been successfully loaded with a wide range of bioactive agents, counting chemotherapeutic drugs, enzymes, nucleic acids, and photosensitizers. Furthermore, the tremendous chemical versatility of CDs offers the possibility of co-delivery of different biomolecules, aiming to create biocompatible theranostic platforms that combine the benefits of several therapeutic strategies towards achieving synergistic results. Moreover, functionalization with targeting ligands has enabled the designed nanocarriers to deliver their freight in a targeted manner, while surface modification with stimuli-responsive moieties ensured a controlled and sustained release at the tumor site.

Summarizing the discussed drug delivery systems, Table 2 gives an overview of the CD-based nanocarriers in a clear and concise manner in correlation to their delivered cargoes and cancer types for which they have been explored.

However, the presented CD-based nanoconstructs are recent formulations, being still in the early testing phases and requiring further investigations before moving to clinical trials. Nonetheless, the promising results obtained by several CD-based nanostructures for cancer management clinically tested in the past two decades [133,134,135] encourage more in-depth research in the field.

## 5. Conclusions

To summarize, the burden of cancer on patients and healthcare systems worldwide compels interdisciplinary research efforts within the global scientific community. As an alternative to conventional therapeutic options for cancer management, the delivery of drugs by complexation with CDs gained significant interest in recent years. Researchers from all around the world have attempted to develop nanovehicles able to carry and release anti-cancer agents in a controlled, sustained, and targeted manner to enhance treatment outcomes while minimizing adverse side effects. These goals could be achieved through CD modification with moieties that either respond to tumor microenvironment triggers or external forces. Thus, a series of nanosystems functionalized with targeting ligands, pH-responsive structures, photolabile compounds, magnetic nanoparticles, and various enzymes have been reported with promising results. CD-based drug delivery systems were tested against many types of cancer, demonstrating encouraging anti-tumor activity in in vitro and in vivo tests.

To conclude, CDs offer tremendous versatility in terms of functionalization and encapsulation possibilities, improve the activity of anti-cancer agents through controlled release and targeted delivery, and provide a safer therapeutic alternative than plain drug administration. Therefore, it can be expected that further investigations in this field could pave the way for the next generation of nanomedicines, envisaging performant solutions for cancer management.

## Figures and Tables

**Table 1:** Main properties of native cyclodextrins (CDs). Created based on information from [34,41,42,43].

	Cyclodextrins	α-CD	β-CD	γ-CD
Properties				
Number of glucopyranose units		6	7	8
Hydrogen donors		18	21	24
Hydrogen acceptors		30	35	40
Molecular weight (Da)		972	1135	1297
Internal diameter (nm)		0.47–0.53	0.60–0.65	0.75–0.83
Outer diameter (nm)		1.46	1.54	1.75
Height of torus (nm)		0.79	0.79	0.79
Internal volume (nm^3^)		0.174	0.262	0.427
Solubility in water (g/100 mL, 25 °C)		14.5	1.85	23.2
Water of hydration (wt %)		10.2	13.2–14.5	8.13–17.7
Number of water molecules taken by cavity		6	11	17

**Table 2:** Summative table of the discussed CD-based drug delivery systems correlated with the targeted types of cancer.

CD-Based Nanocarrier(s)	Anti-Cancer Agent(s)	Type(s) of Cancer	Reference
Disulfide bond-linked permethyl-β-CD	Camptothecin	Lung cancer	[67]
α-CD, β-CD and (2-Hydroxypropyl)-β-CD	Porcupine inhibitor LGK974	Lung cancer	[68]
β-CD coated with PLGA	Erlotinib	Lung cancer	[69]
Sulfobutylether-β-CD	Resveratrol	Lung cancer	[70]
Sulfobutylether-β-CD	Celastrol	Lung cancer	[71]
β-CD polycaprolactone block copolymers conjugated with folic acid	Metformin	Lung cancer	[72]
γ-CD, α-CD, and β-CD	New Zealand propolis	Colorectal adenocarcinoma, colon cancer, gastric cancer, esophageal squamous cell cancer	[75]
Hydroxypropyl-β-CD	Clausenidin	Colon cancer	[77]
β-CD/phillipsite	Oxaliplatin	Colorectal cancer	[76]
Folate-appended methyl-β-CD	Adamantane-grafted hyaluronic acid	Colorectal cancer	[78]
Cationic CD polymers	5-fluorouracil and Interleukin-2	Colorectal cancer	[79]
Folate-targeted polyethylene glycol-modified amphiphilic CD	Ginsenoside Rg3 and quercetin	Colorectal cancer	[80]
Folate-targeted polyethylene glycol-modified amphiphilic cationic CD	Docetaxel and siRNA	Colorectal cancer	[81]
Amphiphilic CDs coated with polyethyleneimine or chitosan	Camptothecin	Colorectal cancer	[82]
Poly(β-CD)	Platinum (IV) prodrug [Pt(IV)-ADA2]	Colorectal cancer	[83]
Folic acid–polyethylene glycol–β- CD	Doxorubicin	Liver cancer	[85]
β-CD modified with lactobionic acid coupled with polyethylene glycol	Doxorubicin	Liver cancer	[86]
β-CD-gated mesoporous silica nanoparticles functionalized with an azobenzene/galactose-grafted polymer	Doxorubicin	Liver cancer	[123]
Folic acid–polyethylene glycol–β- CD	Doxorubicin	Breast cancer	[91]
β-CD polymer	RNA-cleaving DNAzyme targeting c-Myc gene	Breast cancer	[92]
β-CD functionalized dendrimeric graphene oxide-magnetic nanoparticles	Doxorubicin	Breast cancer	[93]
D -α-Tocopherol polyethylene glycol 1000 succinate-modified β-CD	Genistein	Breast cancer	[94]
Folic acid–polyethylene glycol–β- CD	Adamantane and near-infrared fluorophore conjugate	Breast cancer	[95]
Permethyl-β-CD	Meso-tetraphenylporphyrin and meso-tetra(m-hydroxyphenyl)porphyrin	Breast cancer	[96]
β-CD and poly(2-ethyl-2-oxazoline) embedded with iron oxide nanoparticles	Doxorubicin hydrochloride	Breast cancer	[97]
β-CD-chitosan composite	L-asparaginase	Prostate cancer	[101]
β-CD	Carvacrol	Prostate cancer	[102]
β-CD loaded into polylactide stereocomplexed micelles	Doxorubicin	Cervical cancer	[103]
CDs loaded into pegylated liposomes	Pin1 inhibitor	Ovarian cancer	[105]
β-CD	Ticlopidine hydrochloride	Kidney cancer	[108]
Hydroxypropyl-β-CD	Taccalonolide A	Kidney cancer	[109]
CD-based polymers	Camptothecin	Brain cancer	[115]
CDs loaded into liposomes	Butylidenephthalide	Brain cancer	[116]
Hydroxypropyl-β-CD	Disulfiram	Brain cancer	[117]
Cationic CD coated magnetic nanoparticles	Methotrexate	Bone cancer	[120]
Magnetic hyperbranched β-CD	Doxorubicin	Bone cancer	[121]
Polyethylenimine–bisphosphonate–CD ternary conjugates	Doxorubicin	Bone cancer	[122]
Hydroxypropyl-β-CD	Saikosaponin-d	Skin cancer	[128]
β-CD, γ-CD, and γ-CD functionalized with arginine-glycine-aspartic or arginine moieties	Doxorubicin and oxaliplatin	Lung cancer, liver cancer	[16]
β-CD	Acetylshikonin	Colorectal cancer, breast adenocarcinoma	[127]

## Data Availability

Not applicable.

## References

[1] Sung H., Ferlay J., Siegel R.L., Laversanne M., Soerjomataram I., Jemal A., Bray F. (2021). Global Cancer Statistics 2020: GLOBOCAN Estimates of Incidence and Mortality Worldwide for 36 Cancers in 185 Countries. CA A Cancer J. Clin..

[2] Gadade D.D., Rathi P.B., Sangshetti J.N., Kulkarni D.A., Venkatesan J., Kim S.-K., Anil S.P.D.R. (2022). 18—Multifunctional cyclodextrin nanoparticles: A promising theranostic tool for strategic targeting of cancer. Polysaccharide Nanoparticles.

[3] Tian B., Hua S., Liu J. (2020). Cyclodextrin-based delivery systems for chemotherapeutic anticancer drugs: A review. Carbohydr. Polym..

[4] Zhang D., Lv P., Zhou C., Zhao Y., Liao X., Yang B. (2019). Cyclodextrin-based delivery systems for cancer treatment. Mater. Sci. Eng. C.

[5] World Health Organisation (2020). Assessing National Capacity for the Prevention and Control of Noncommunicable Diseases: Report of the 2019 Global Survey.

[6] Garg P. (2021). Selective Preference of Antibody Mimetics over Antibody, as Binding Molecules, for Diagnostic and Therapeutic Applications in Cancer Therapy. Biointerface Res. Appl. Chem..

[7] Mansouri F. (2021). Role of Telemedicine and Telegenetics Framework for the Management of Cancer Patients During the COVID-19 Pandemic. Biointerface Res. Appl. Chem..

[8] Murtaza M., Baig M.M.A., Ahmed J., Serbanoiu L.I., Busnatu S.S. (2022). Higher Mortality Associated With New-Onset Atrial Fibrillation in Cancer Patients: A Systematic Review and Meta-Analysis. Front. Cardiovasc. Med..

[9] Upadhyay A. (2021). Cancer: An unknown territory; rethinking before going ahead. Genes Dis..

[10] Busnatu Ș., Niculescu A.-G., Bolocan A., Petrescu G.E.D., Păduraru D.N., Năstasă I., Lupușoru M., Geantă M., Andronic O., Grumezescu A.M. (2022). Clinical Applications of Artificial Intelligence—An Updated Overview. J. Clin. Med..

[11] Du Y., Chen B. (2019). Combination of drugs and carriers in drug delivery technology and its development. Drug Des. Dev. Ther..

[12] Tran P., Lee S.-E., Kim D.-H., Pyo Y.-C., Park J.-S. (2020). Recent advances of nanotechnology for the delivery of anticancer drugs for breast cancer treatment. J. Pharm. Investig..

[13] Fahmy S.A., Brüßler J., Alawak M., El-Sayed M.M.H., Bakowsky U., Shoeib T. (2019). Chemotherapy Based on Supramolecular Chemistry: A Promising Strategy in Cancer Therapy. Pharmaceutics.

[14] Chinnamanyakar R., Ramanathan E.M. (2021). Anti-cancer and Antimicrobial Activity, In-Silico ADME and Docking Studies of Biphenyl Pyrazoline Derivatives. Biointerface Res. Appl. Chem..

[15] Das M., Solanki A., Joshi A., Devkar R., Seshadri S., Thakore S. (2019). β-cyclodextrin based dual-responsive multifunctional nanotheranostics for cancer cell targeting and dual drug delivery. Carbohydr. Polym..

[16] Bognanni N., Viale M., Distefano A., Tosto R., Bertola N., Loiacono F., Ponassi M., Spinelli D., Pappalardo G., Vecchio G. (2021). Cyclodextrin Polymers as Delivery Systems for Targeted Anti-Cancer Chemotherapy. Molecules.

[17] Alphandéry E. (2020). Natural Metallic Nanoparticles for Application in Nano-Oncology. Int. J. Mol. Sci..

[18] Ion D., Niculescu A.-G., Păduraru D.N., Andronic O., Mușat F., Grumezescu A.M., Bolocan A. (2022). An Up-to-Date Review of Natural Nanoparticles for Cancer Management. Pharmaceutics.

[19] Aiello P., Consalvi S., Poce G., Raguzzini A., Toti E., Palmery M., Biava M., Bernardi M., Kamal M.A., Perry G. (2021). Dietary flavonoids: Nano delivery and nanoparticles for cancer therapy. Semin. Cancer Biol..

[20] De la Torre P., Pérez-Lorenzo M.J., Alcázar-Garrido Á., Flores A.I. (2020). Cell-Based Nanoparticles Delivery Systems for Targeted Cancer Therapy: Lessons from Anti-Angiogenesis Treatments. Molecules.

[21] Niculescu A.-G., Grumezescu A.M. (2022). Novel Tumor-Targeting Nanoparticles for Cancer Treatment—A Review. Int. J. Mol. Sci..

[22] Bakirhan N.K., Tok T.T., Ozkan S.A. (2019). The redox mechanism investigation of non-small cell lung cancer drug: Erlotinib via theoretical and experimental techniques and its host–guest detection by β-Cyclodextrin nanoparticles modified glassy carbon electrode. Sens. Actuators B Chem..

[23] Sivakumar M.P., Peimanfard S., Zarrabi A., Khosravi A., Islami M. (2020). Cyclodextrin-Based Nanosystems as Drug Carriers for Cancer Therapy. Anti-Cancer Agents Med. Chem..

[24] Yousefi M., Shadnoush M., Sohrabvandi S., Khorshidian N., Mortazavian A.M. (2021). Encapsulation Systems for Delivery of Flavonoids: A Review. Biointerface Res. Appl. Chem..

[25] Kanojia N., Singh S., Singh J., Sharma N., Grewal A.S., Rani L., Thapa K., Arora S. (2021). Recent Advancements and Applications of Inhalable Microparticles Based Drug Delivery Systems in Respiratory Disorders. Biointerface Res. Appl. Chem..

[26] Halavach T.M., Savchuk E.S., Bobovich A.S., Dudchik N.V., Tsygankow V.G., Tarun E.I., Yantsevich A.V., Kurchenko V.P., Kharitonov V.D., Asafov V.A. (2021). Antimutagenic and Antibacterial Activity of beta-Cyclodextrin Clathrates with Extensive Hydrolysates of Colostrum and Whey. Biointerface Res. Appl. Chem..

[27] Cid-Samamed A., Rakmai J., Mejuto J.C., Simal-Gandara J., Astray G. (2022). Cyclodextrins inclusion complex: Preparation methods, analytical techniques and food industry applications. Food Chem..

[28] Mousazadeh H., Pilehvar-Soltanahmadi Y., Dadashpour M., Zarghami N. (2021). Cyclodextrin based natural nanostructured carbohydrate polymers as effective non-viral siRNA delivery systems for cancer gene therapy. J. Control. Release.

[29] Kfoury M., Landy D., Fourmentin S. (2018). Characterization of Cyclodextrin/Volatile Inclusion Complexes: A Review. Molecules.

[30] Kost B., Brzeziński M., Socka M., Baśko M., Biela T. (2020). Biocompatible Polymers Combined with Cyclodextrins: Fascinating Materials for Drug Delivery Applications. Molecules.

[31] Zhang X., Ma X., Wang K., Lin S., Zhu S., Dai Y., Xia F. (2018). Recent Advances in Cyclodextrin-Based Light-Responsive Supramolecular Systems. Macromol. Rapid Commun..

[32] Radu C.-D., Parteni O., Ochiuz L. (2016). Applications of cyclodextrins in medical textiles. J. Control. Release.

[33] Del Valle E.M. (2004). Cyclodextrins and their uses: A review. Process Biochem..

[34] Sengupta P.K., Bhattacharjee S., Chakraborty S., Bhowmik S., Grumezescu A.M. (2018). Chapter 15—Encapsulation of pharmaceutically active dietary polyphenols in cyclodextrin-based nanovehicles: Insights from spectroscopic studies. Design of Nanostructures for Versatile Therapeutic Applications.

[35] Zafar N., Fessi H., Elaissari A. (2014). Cyclodextrin containing biodegradable particles: From preparation to drug delivery applications. Int. J. Pharm..

[36] Muankaew C., Loftsson T. (2018). Cyclodextrin-based formulations: A non-invasive platform for targeted drug delivery. Basic Clin. Pharmacol. Toxicol..

[37] Crini G., Fourmentin S., Fenyvesi É., Torri G., Fourmentin M., Morin-Crini N. (2018). Fundamentals and applications of cyclodextrins. Cyclodextrin Fundamentals, Reactivity and Analysis.

[38] Řezanka M. (2018). Synthesis of cyclodextrin derivatives. Cyclodextrin Fundamentals, Reactivity and Analysis.

[39] Leclercq L. (2016). Interactions between cyclodextrins and cellular components: Towards greener medical applications?. Beilstein J. Org. Chem..

[40] Leclercq L. (2016). Smart medical textiles based on cyclodextrins for curative or preventive patient care. Active Coatings for Smart Textiles.

[41] Gharibzahedi S.M.T., Jafari S.M., Jafari S.M. (2017). 7—Nanocapsule formation by cyclodextrins. Nanoencapsulation Technologies for the Food and Nutraceutical Industries.

[42] Jambhekar S.S., Breen P. (2016). Cyclodextrins in pharmaceutical formulations I: Structure and physicochemical properties, formation of complexes, and types of complex. Drug Discov. Today.

[43] Saokham P., Muankaew C., Jansook P., Loftsson T. (2018). Solubility of Cyclodextrins and Drug/Cyclodextrin Complexes. Molecules.

[44] Haley R.M., Gottardi R., Langer R., Mitchell M.J. (2020). Cyclodextrins in drug delivery: Applications in gene and combination therapy. Drug Deliv. Transl. Res..

[45] Carneiro S.B., Duarte C., Ílary F., Heimfarth L., Quintans S., de Souza J., Quintans-Júnior L.J., Veiga Júnior V.F.d., Neves de Lima Á.A. (2019). Cyclodextrin–drug inclusion complexes: In vivo and in vitro approaches. Int. J. Mol. Sci..

[46] Wimmer T. (2000). Cyclodextrins. Ullmann’s Encyclopedia of Industrial Chemistry.

[47] Raut S.Y., Manne A.S.N., Kalthur G., Jain S., Mutalik S. (2019). Cyclodextrins as Carriers in Targeted Delivery of Therapeutic Agents: Focused Review on Traditional and Inimitable Applications. Curr. Pharm. Des..

[48] Santos A.C., Costa D., Ferreira L., Guerra C., Pereira-Silva M., Pereira I., Peixoto D., Ferreira N.R., Veiga F. (2021). Cyclodextrin-based delivery systems for in vivo-tested anticancer therapies. Drug Deliv. Transl. Res..

[49] Kaur C., Garg U. (2021). Artificial intelligence techniques for cancer detection in medical image processing: A review. Mater. Today Proc..

[50] Mattiuzzi C., Lippi G. (2019). Current Cancer Epidemiology. J. Epidemiol. Glob. Health.

[51] Siegel R.L., Miller K.D., Fuchs H.E., Jemal A. (2022). Cancer statistics, 2022. CA Cancer J. Clin..

[52] Madamsetty V.S., Mukherjee A., Mukherjee S. (2019). Recent trends of the bio-inspired nanoparticles in cancer theranostics. Front. Pharmacol..

[53] Choi D.G., Venkatesan J., Shim M.S. (2019). Selective Anticancer Therapy Using Pro-Oxidant Drug-Loaded Chitosan–Fucoidan Nanoparticles. Int. J. Mol. Sci..

[54] Abdelaziz H.M., Gaber M., Abd-Elwakil M.M., Mabrouk M.T., Elgohary M.M., Kamel N.M., Kabary D.M., Freag M.S., Samaha M.W., Mortada S.M. (2018). Inhalable particulate drug delivery systems for lung cancer therapy: Nanoparticles, microparticles, nanocomposites and nanoaggregates. J. Control. Release.

[55] Yagawa Y., Tanigawa K., Kobayashi Y., Yamamoto M. (2017). Cancer immunity and therapy using hyperthermia with immunotherapy, radiotherapy, chemotherapy, and surgery. J. Cancer Metastasis Treat..

[56] Amjad M.T., Chidharla A., Kasi A. (2020). Cancer Chemotherapy. StatPearls. https://www.ncbi.nlm.nih.gov/books/NBK564367/.

[57] Alshehri A.A., Almughem F.A., Aldossary A.M., Tawfik E.A., Al-Fahad A.J., Alyahya S., Alomary M.N., Ansari M.A., Rehman S. (2021). Microbial Nanoparticles for Cancer Treatment. Microbial Nanotechnology: Green Synthesis and Applications.

[58] Haag R. (2004). Supramolecular Drug-Delivery Systems Based on Polymeric Core–Shell Architectures. Angew. Chem. Int. Ed..

[59] Haag R., Kratz F. (2006). Polymer Therapeutics: Concepts and Applications. Angew. Chem. Int. Ed..

[60] Lungu I.I., Grumezescu A.M., Volceanov A., Andronescu E. (2019). Nanobiomaterials Used in Cancer Therapy: An Up-To-Date Overview. Molecules.

[61] Alavi M., Nokhodchi A. (2022). Micro- and nanoformulations of paclitaxel based on micelles, liposomes, cubosomes, and lipid nanoparticles: Recent advances and challenges. Drug Discov. Today.

[62] Menezes P.D.P., Andrade T.A., Frank L.A., de Souza E., Trindade G., Trindade I.A.S., Serafini M.R., Guterres S.S., Araújo A.A.S. (2019). Advances of nanosystems containing cyclodextrins and their applications in pharmaceuticals. Int. J. Pharm..

[63] Qiu N., Li X., Liu J. (2017). Application of cyclodextrins in cancer treatment. J. Incl. Phenom. Macrocycl. Chem..

[64] Barta J.A., Powell C.A., Wisnivesky J.P. (2019). Global Epidemiology of Lung Cancer. Ann. Glob. Health.

[65] Howlader N., Forjaz G., Mooradian M.J., Meza R., Kong C.Y., Cronin K.A., Mariotto A.B., Lowy D.R., Feuer E.J. (2020). The effect of advances in lung-cancer treatment on population mortality. N. Engl. J. Med..

[66] Mohammed A., Makia R., Ali M., Raheem R., Yousif E. (2021). Cytotoxic Effects of Valsartan Organotin(IV) Complexes on Human Lung Cancer Cells. Biointerface Res. Appl. Chem..

[67] Dai X., Zhang B., Zhou W., Liu Y. (2020). High-Efficiency Synergistic Effect of Supramolecular Nanoparticles Based on Cyclodextrin Prodrug on Cancer Therapy. Biomacromolecules.

[68] Guimaraes P.P.G., Tan M., Tammela T., Wu K., Chung A., Oberli M., Wang K., Spektor R., Riley R.S., Viana C.T.R. (2018). Potent in vivo lung cancer Wnt signaling inhibition via cyclodextrin-LGK974 inclusion complexes. J. Control. Release.

[69] Vaidya B., Parvathaneni V., Kulkarni N.S., Shukla S.K., Damon J.K., Sarode A., Kanabar D., Garcia J.V., Mitragotri S., Muth A. (2019). Cyclodextrin modified erlotinib loaded PLGA nanoparticles for improved therapeutic efficacy against non-small cell lung cancer. Int. J. Biol. Macromol..

[70] Wang X., Parvathaneni V., Shukla S.K., Kulkarni N.S., Muth A., Kunda N.K., Gupta V. (2020). Inhalable resveratrol-cyclodextrin complex loaded biodegradable nanoparticles for enhanced efficacy against non-small cell lung cancer. Int. J. Biol. Macromol..

[71] Shukla S.K., Chan A., Parvathaneni V., Kanabar D.D., Patel K., Ayehunie S., Muth A., Gupta V. (2020). Enhanced solubility, stability, permeation and anti-cancer efficacy of Celastrol-β-cyclodextrin inclusion complex. J. Mol. Liq..

[72] Lin X., Bai Y., Jiang Q. (2022). Precise fabrication of folic acid-targeted therapy on metformin encapsulated β-cyclodextrin nanomaterials for treatment and care of lung cancer. Process Biochem..

[73] Arnold M., Abnet C.C., Neale R.E., Vignat J., Giovannucci E.L., McGlynn K.A., Bray F. (2020). Global Burden of 5 Major Types of Gastrointestinal Cancer. Gastroenterology.

[74] Shahiwala A. (2020). Cyclodextrin conjugates for colon drug delivery. J. Drug Deliv. Sci. Technol..

[75] Catchpole O., Mitchell K., Bloor S., Davis P., Suddes A. (2018). Anti-gastrointestinal cancer activity of cyclodextrin-encapsulated propolis. J. Funct. Foods.

[76] Altoom N., Ibrahim S.M., Othman S.I., Allam A.A., Alqhtani H.A., Al-Otaibi F.S., Abukhadra M.R. (2022). Characterization of β-cyclodextrin/phillipsite (β-CD/Ph) composite as a potential carrier for oxaliplatin as therapy for colorectal cancer; loading, release, and cytotoxicity. Colloids Surf. Physicochem. Eng. Asp..

[77] Al-Abboodi A.S., Al-Sheikh W.a.M., Eid E.E.M., Azam F., Al-Qubaisi M.S. (2021). Inclusion complex of clausenidin with hydroxypropyl-β-cyclodextrin: Improved physicochemical properties and anti-colon cancer activity. Saudi Pharm. J..

[78] Elamin K.M., Motoyama K., Higashi T., Yamashita Y., Tokuda A., Arima H. (2018). Dual targeting system by supramolecular complex of folate-conjugated methyl-β-cyclodextrin with adamantane-grafted hyaluronic acid for the treatment of colorectal cancer. Int. J. Biol. Macromol..

[79] Akkın S., Varan G., Aksüt D., Malanga M., Ercan A., Şen M., Bilensoy E. (2022). A different approach to immunochemotherapy for colon Cancer: Development of nanoplexes of cyclodextrins and Interleukin-2 loaded with 5-FU. Int. J. Pharm..

[80] Sun D., Zou Y., Song L., Han S., Yang H., Chu D., Dai Y., Ma J., O’Driscoll C.M., Yu Z. (2022). A cyclodextrin-based nanoformulation achieves co-delivery of ginsenoside Rg3 and quercetin for chemo-immunotherapy in colorectal cancer. Acta Pharm. Sin. B.

[81] Zou Y., Xiao F., Song L., Sun B., Sun D., Chu D., Wang L., Han S., Yu Z., O’Driscoll C.M. (2021). A folate-targeted PEGylated cyclodextrin-based nanoformulation achieves co-delivery of docetaxel and siRNA for colorectal cancer. Int. J. Pharm..

[82] Ünal S., Aktaş Y., Benito J.M., Bilensoy E. (2020). Cyclodextrin nanoparticle bound oral camptothecin for colorectal cancer: Formulation development and optimization. Int. J. Pharm..

[83] Zhang R., You X., Luo M., Zhang X., Fang Y., Huang H., Kang Y., Wu J. (2022). Poly(β-cyclodextrin)/platinum prodrug supramolecular nano system for enhanced cancer therapy: Synthesis and in vivo study. Carbohydr. Polym..

[84] Rumgay H., Ferlay J., de Martel C., Georges D., Ibrahim A.S., Zheng R., Wei W., Lemmens V.E.P.P., Soerjomataram I. (2022). Global, regional and national burden of primary liver cancer by subtype. Eur. J. Cancer.

[85] Fan W., Xu Y., Li Z., Li Q. (2019). Folic acid-modified β-cyclodextrin nanoparticles as drug delivery to load DOX for liver cancer therapeutics. Soft Mater..

[86] Yang T., Du G., Cui Y., Yu R., Hua C., Tian W., Zhang Y. (2019). pH-sensitive doxorubicin-loaded polymeric nanocomplex based on β-cyclodextrin for liver cancer-targeted therapy. Int. J. Nanomed..

[87] Ahmad A., Ahmad A. (2019). Breast Cancer Statistics: Recent Trends. Breast Cancer Metastasis and Drug Resistance: Challenges and Progress.

[88] Cao W., Chen H.-D., Yu Y.-W., Li N., Chen W.-Q., Ni J. (2021). Changing profiles of cancer burden worldwide and in China: A secondary analysis of the global cancer statistics 2020. Chin. Med. J..

[89] Grewal I.K., Singh S., Arora S., Sharma N. (2021). Polymeric nanoparticles for breast cancer therapy: A comprehensive review. Biointerface Res. Appl. Chem.

[90] Aghazadeh T., Bakhtiari N., Rad I.A., Ramezani F. (2021). Formulation of Kaempferol in Nanostructured Lipid Carriers (NLCs): A Delivery Platform to Sensitization of MDA-MB468 Breast Cancer Cells to Paclitaxel. Biointerface Res. Appl. Chem..

[91] Hyun H., Lee S., Lim W., Jo D., Jung J.S., Jo G., Kim S.Y., Lee D.-W., Um S., Yang D.H. (2019). Engineered beta-cyclodextrin-based carrier for targeted doxorubicin delivery in breast cancer therapy in vivo. J. Ind. Eng. Chem..

[92] Farrokhi F., Karami Z., Esmaeili-Mahani S., Heydari A. (2018). Delivery of DNAzyme targeting c-Myc gene using β-cyclodextrin polymer nanocarrier for therapeutic application in human breast cancer cell line. J. Drug Deliv. Sci. Technol..

[93] Mihanfar A., Targhazeh N., Sadighparvar S., Darband S.G., Majidinia M., Yousefi B. (2021). Doxorubicin loaded magnetism nanoparticles based on cyclodextrin dendritic-graphene oxide inhibited MCF-7 cell proliferation. Biomol. Concepts.

[94] Zafar A., Alruwaili N.K., Imam S.S., Alsaidan O.A., Alharbi K.S., Mostafa E.M., Musa A., Gilani S.J., Ghoneim M.M., Alshehri S. (2022). Formulation of ternary genistein β-cyclodextrin inclusion complex: In vitro characterization and cytotoxicity assessment using breast cancer cell line. J. Drug Deliv. Sci. Technol..

[95] Lee D.-W., Jo J., Jo D., Kim J., Min J.-J., Yang D.H., Hyun H. (2018). Supramolecular assembly based on host–guest interaction between beta-cyclodextrin and adamantane for specifically targeted cancer imaging. J. Ind. Eng. Chem..

[96] Panagiotakis S., Mavroidi B., Athanasopoulos A., Charalambidis G., Coutsolelos A.G., Paravatou-Petsotas M., Pelecanou M., Mavridis I.M., Yannakopoulou K. (2022). Unsymmetrical, monocarboxyalkyl meso-arylporphyrins in the photokilling of breast cancer cells using permethyl-β-cyclodextrin as sequestrant and cell uptake modulator. Carbohydr. Polym..

[97] Soleimani K., Arkan E., Derakhshankhah H., Haghshenas B., Jahanban-Esfahlan R., Jaymand M. (2021). A novel bioreducible and pH-responsive magnetic nanohydrogel based on β-cyclodextrin for chemo/hyperthermia therapy of cancer. Carbohydr. Polym..

[98] Ercan A., Çelebier M., Varan G., Öncül S., Nenni M., Kaplan O., Bilensoy E. (2018). Global omics strategies to investigate the effect of cyclodextrin nanoparticles on MCF-7 breast cancer cells. Eur. J. Pharm. Sci..

[99] Kasinathan K., Marimuthu K., Murugesan B., Pandiyan N., Pandi B., Mahalingam S., Selvaraj B. (2021). Cyclodextrin functionalized multi-layered MoS2 nanosheets and its biocidal activity against pathogenic bacteria and MCF-7 breast cancer cells: Synthesis, characterization and in-vitro biomedical evaluation. J. Mol. Liq..

[100] Peremiquel-Trillas P., Frias-Gomez J., Alemany L., Ameijide A., Vilardell M., Marcos-Gragera R., Paytubi S., Ponce J., Martínez J.M., Pineda M. (2022). Predicting Ovarian-Cancer Burden in Catalonia by 2030: An Age–Period–Cohort Modelling. Int. J. Environ. Res. Public Health.

[101] Baskar G., Supria Sree N. (2020). Synthesis, characterization and anticancer activity of β-cyclodextrin-Asparaginase nanobiocomposite on prostate and lymphoma cancer cells. J. Drug Deliv. Sci. Technol..

[102] Trindade G.G.G., Thrivikraman G., Menezes P.P., França C.M., Lima B.S., Carvalho Y.M.B.G., Souza E.P.B.S.S., Duarte M.C., Shanmugam S., Quintans-Júnior L.J. (2019). Carvacrol/β-cyclodextrin inclusion complex inhibits cell proliferation and migration of prostate cancer cells. Food Chem. Toxicol..

[103] Kost B., Brzeziński M., Cieślak M., Królewska-Golińska K., Makowski T., Socka M., Biela T. (2019). Stereocomplexed micelles based on polylactides with β-cyclodextrin core as anti-cancer drug carriers. Eur. Polym. J..

[104] Reis C.A., Rodrigues C.F., Moreira A.F., Jacinto T.A., Ferreira P., Correia I.J. (2019). Development of gold-core silica shell nanospheres coated with poly-2-ethyl-oxazoline and β-cyclodextrin aimed for cancer therapy. Mater. Sci. Eng. C.

[105] Russo Spena C., De Stefano L., Palazzolo S., Salis B., Granchi C., Minutolo F., Tuccinardi T., Fratamico R., Crotti S., D’Aronco S. (2018). Liposomal delivery of a Pin1 inhibitor complexed with cyclodextrins as new therapy for high-grade serous ovarian cancer. J. Control. Release.

[106] Linehan W.M., Schmidt L.S., Crooks D.R., Wei D., Srinivasan R., Lang M., Ricketts C.J. (2019). The Metabolic Basis of Kidney Cancer. Cancer Discov..

[107] Peired A.J., Campi R., Angelotti M.L., Antonelli G., Conte C., Lazzeri E., Becherucci F., Calistri L., Serni S., Romagnani P. (2021). Sex and Gender Differences in Kidney Cancer: Clinical and Experimental Evidence. Cancers.

[108] Bomzan P., Roy N., Rai V., Roy D., Ghosh S., Kumar A., Roy K., Chakrabarty R., Das J., Dakua V.K. (2022). Inclusion of an antiplatelet agent inside into β-cyclodextrin for biochemical applications with diverse authentications. Food Chem. Adv..

[109] Han J., Zhang S., Niu J., Zhang C., Dai W., Wu Y., Hu L. (2020). Development of Taccalonolide AJ-Hydroxypropyl-β-Cyclodextrin Inclusion Complexes for Treatment of Clear Cell Renal-Cell Carcinoma. Molecules.

[110] Teleanu R.I., Preda M.D., Niculescu A.-G., Vladâcenco O., Radu C.I., Grumezescu A.M., Teleanu D.M. (2022). Current Strategies to Enhance Delivery of Drugs across the Blood–Brain Barrier. Pharmaceutics.

[111] Ayub A., Wettig S. (2022). An Overview of Nanotechnologies for Drug Delivery to the Brain. Pharmaceutics.

[112] Zottel A., Videtič Paska A., Jovčevska I. (2019). Nanotechnology Meets Oncology: Nanomaterials in Brain Cancer Research, Diagnosis and Therapy. Materials.

[113] Teleanu D.M., Negut I., Grumezescu V., Grumezescu A.M., Teleanu R.I. (2019). Nanomaterials for Drug Delivery to the Central Nervous System. Nanomaterials.

[114] Asaduzzaman S., Chakma R., Rehana H., Raihan M. (2021). Regulatory Gene Network Pathway among Brain Cancer and Associated Disease: A Computational Analysis. Biointerface Res. Appl. Chem..

[115] Chen Y.-F., Wang Y.-H., Lei C.-S., Changou C.A., Davis M.E., Yen Y. (2019). Host immune response to anti-cancer camptothecin conjugated cyclodextrin-based polymers. J. Biomed. Sci..

[116] Lin E.-Y., Chen Y.-S., Li Y.-S., Chen S.-R., Lee C.-H., Huang M.-H., Chuang H.-M., Harn H.-J., Yang H.-H., Lin S.-Z. (2020). Liposome Consolidated with Cyclodextrin Provides Prolonged Drug Retention Resulting in Increased Drug Bioavailability in Brain. Int. J. Mol. Sci..

[117] Qu Y., Sun X., Ma L., Li C., Xu Z., Ma W., Zhou Y., Zhao Z., Ma D. (2021). Therapeutic effect of disulfiram inclusion complex embedded in hydroxypropyl-β-cyclodextrin on intracranial glioma-bearing male rats via intranasal route. Eur. J. Pharm. Sci..

[118] Ferguson J.L., Turner S.P. (2018). Bone cancer: Diagnosis and treatment principles. Am. Fam. Physician.

[119] Bădilă A.E., Rădulescu D.M., Niculescu A.-G., Grumezescu A.M., Rădulescu M., Rădulescu A.R. (2021). Recent Advances in the Treatment of Bone Metastases and Primary Bone Tumors: An Up-to-Date Review. Cancers.

[120] Ahmadi D., Zarei M., Rahimi M., Khazaie M., Asemi Z., Mir S.M., Sadeghpour A., Karimian A., Alemi F., Rahmati-Yamchi M. (2020). Preparation and in-vitro evaluation of pH-responsive cationic cyclodextrin coated magnetic nanoparticles for delivery of methotrexate to the Saos-2 bone cancer cells. J. Drug Deliv. Sci. Technol..

[121] Khelghati N., Rasmi Y., Farahmandan N., Sadeghpour A., Mir S.M., Karimian A., Yousefi B. (2020). Hyperbranched polyglycerol β-cyclodextrin as magnetic platform for optimization of doxorubicin cytotoxic effects on Saos-2 bone cancerous cell line. J. Drug Deliv. Sci. Technol..

[122] Plesselova S., Garcia-Cerezo P., Blanco V., Reche-Perez F.J., Hernandez-Mateo F., Santoyo-Gonzalez F., Giron-Gonzalez M.D., Salto-Gonzalez R. (2021). Polyethylenimine–Bisphosphonate–Cyclodextrin Ternary Conjugates: Supramolecular Systems for the Delivery of Antineoplastic Drugs. J. Med. Chem..

[123] Wu Y., Xu Z., Sun W., Yang Y., Jin H., Qiu L., Chen J., Chen J. (2019). Co-responsive smart cyclodextrin-gated mesoporous silica nanoparticles with ligand-receptor engagement for anti-cancer treatment. Mater. Sci. Eng. C.

[124] Mousazadeh H., Bonabi E., Zarghami N. (2022). Stimulus-responsive drug/gene delivery system based on polyethylenimine cyclodextrin nanoparticles for potential cancer therapy. Carbohydr. Polym..

[125] Jia D., Ma X., Lu Y., Li X., Hou S., Gao Y., Xue P., Kang Y., Xu Z. (2021). ROS-responsive cyclodextrin nanoplatform for combined photodynamic therapy and chemotherapy of cancer. Chin. Chem. Lett..

[126] Pooresmaeil M., Namazi H. (2018). β-Cyclodextrin grafted magnetic graphene oxide applicable as cancer drug delivery agent: Synthesis and characterization. Mater. Chem. Phys..

[127] Vukic M.D., Vukovic N.L., Popovic S.L., Todorovic D.V., Djurdjevic P.M., Matic S.D., Mitrovic M.M., Popovic A.M., Kacaniova M.M., Baskic D.D. (2020). Effect of β-cyclodextrin encapsulation on cytotoxic activity of acetylshikonin against HCT-116 and MDA-MB-231 cancer cell lines. Saudi Pharm. J..

[128] Hu S.C.-S., Lai Y.-C., Lin C.-L., Tzeng W.-S., Yen F.-L. (2019). Inclusion complex of saikosaponin-d with hydroxypropyl-β-cyclodextrin: Improved physicochemical properties and anti-skin cancer activity. Phytomedicine.

[129] Parvathaneni V., Elbatanony R.S., Shukla S.K., Kulkarni N.S., Kanabar D.D., Chauhan G., Ayehunie S., Chen Z.-S., Muth A., Gupta V. (2021). Bypassing P-glycoprotein mediated efflux of afatinib by cyclodextrin complexation—Evaluation of intestinal absorption and anti-cancer activity. J. Mol. Liq..

[130] Xu J., Ren X., Guo T., Sun X., Chen X., Patterson L.H., Li H., Zhang J. (2019). NLG919/cyclodextrin complexation and anti-cancer therapeutic benefit as a potential immunotherapy in combination with paclitaxel. Eur. J. Pharm. Sci..

[131] Rodell C.B., Arlauckas S.P., Cuccarese M.F., Garris C.S., Li R., Ahmed M.S., Kohler R.H., Pittet M.J., Weissleder R. (2018). TLR7/8-agonist-loaded nanoparticles promote the polarization of tumour-associated macrophages to enhance cancer immunotherapy. Nat. Biomed. Eng..

[132] Zhang Y., Zhang N., Yao S., Yao C., Li Y., Ke M., Zhang S., Qian L., Hu X., Ren F. (2022). Cyclodextrin single isomer-based vesicle for chlorin e6 delivery and enhanced efficiency of photodynamic therapy for cancer treatment. J. Mol. Liq..

[133] Topotecan Hydrochloride or Cyclodextrin-Based Polymer-Camptothecin CRLX101 in Treating Patients with Recurrent Small Cell Lung Cancer. https://www.clinicaltrials.gov/ct2/show/results/NCT01803269?term=cyclodextrin&cond=cancer&draw=2&rank=1.

[134] Sputum Labeling Utilizing Synthetic Meso-Tetra (4-Carboxyphenyl) Porphyrin (TCPP) for Detection of Lung Cancer. https://www.clinicaltrials.gov/ct2/show/study/NCT03837600?term=cyclodextrin&cond=cancer&draw=2&rank=4.

[135] Pilot Trial of CRLX101 in Treatment of Patients with Advanced or Metastatic Stomach, Gastroesophageal, or Esophageal Cancer That Cannot be Removed by Surgery. https://www.clinicaltrials.gov/ct2/show/study/NCT01612546?term=cyclodextrin&cond=cancer&draw=2&rank=5.

